# Silent changes in taxonomic, functional and phylogenetic diversity of birds in Qiyunshan National Nature Reserve, south China

**DOI:** 10.3897/BDJ.13.e145093

**Published:** 2025-03-25

**Authors:** Binqiang Li, Jian Lu, Xin Zhong, Daohan Li, Bailin Li, Nehafta Bibi, Kechuan Linghu, Shanjun Ma, Pinghua Zhong

**Affiliations:** 1 College of Forestry, Southwest Forestry University, Kunming, China College of Forestry, Southwest Forestry University Kunming China; 2 Planing and Design Institute, Yunnan Forestry Technological College, Kunming, China Planing and Design Institute, Yunnan Forestry Technological College Kunming China; 3 Jiangxi Environmental Engineering Vocational College, Ganzhou, China Jiangxi Environmental Engineering Vocational College Ganzhou China; 4 Jiangxi Qiyunshan National Nature Reserve Administration, Ganzhou, China Jiangxi Qiyunshan National Nature Reserve Administration Ganzhou China

**Keywords:** temporal beta diversity, community change, functional diversity, community assembly, Qiyunshan National Nature Reserve, ecosystem stability, conservation effectiveness evaluation

## Abstract

Temporal taxonomic shifts have been documented in bird communities within protected areas. However, the potential impact of these changes on functional diversity and phylogenetic diversity remains poorly understood. In this study, we monitored bird communities in Qiyunshan National Nature Reserve in southern China for nine years (2014-2022). We examined temporal trends in taxonomic, functional and phylogenetic diversity metrics and compared observed phylogenetic diversity values with expected values to determine the mechanisms driving community assembly. Additionally, we evaluated the temporal trend of beta diversity. A total of 118 bird species were recorded, with the dominant species including Chestnut Bulbul (*Hemixoscastanonotus*), Grey-cheeked Fulvetta (*Alcippemorrisonia*) and Great Tit (*Parusmajor*). We found that species turnover was the principal driver of temporal variations in species composition. However, species richness, functional diversity and phylogenetic diversity fluctuated throughout the study period, showing no clear trend of increase or decrease. Our findings indicate that the composition of bird communities is shaped by environmental filtering and neutral processes. The changes in taxonomics may be due to changes in the availability of resources and random substitution arising from the dispersion process. Protected areas have the potential to attract new bird species with similar functional and genetic relationships to those already present. This leads to minimal changes to overall functional and phylogenetic diversity, suggesting a degree of functional redundancy amongst species that are replaced or added. Notably, we observed a persistent increase in species loss over time, raising concerns about the potential impact on the future functional stability of the system. We highlight that the asynchronous patterns of taxonomic, functional and phylogenetic diversity in birds emphasise the importance of multidimensional diversity metrics. Consequently, we suggest that functional and phylogenetic diversity should be regarded as essential indicators alongside species richness when evaluating conservation outcomes in nature reserves. This approach provides a more comprehensive understanding of the complexity of ecological communities and provides information for more effective conservation strategies.

## Introduction

Biodiversity loss is a major environmental concern, with alarming declines observed in many species worldwide. For instance, during the 1970s, North America was home to around 529 bird species totalling nearly 3 billion individuals (Rosenberg et al. 2019). However, over the past 48 years, 303 species have experienced population declines, resulting in an overall loss of about 29% in total abundance (Rosenberg et al. 2019). In South Africa, 50% of bird species that rely on forests are experiencing reductions in their ranges, with the most significant losses occurring in the eastern Cape Province (Cooper et al. 2017). Similar declines have been observed in Asia (Xu et al. 2017), Australia (Lindenmayer et al. 2018, Woodworth et al. 2021) and Europe (Lehikoinen et al. 2018). However, the ecological consequences of these decline and their impact on ecosystem services remain inadequately understood (Larsen et al. 2024). A notable deficiency in research exists that explores the trends in phylogenetic and functional diversity patterns associated with declines in species richness (Che et al. 2021). A conservation challenge may emerge from the discrepancies amongst taxonomic diversity (TD), functional diversity (FD) and phylogenetic diversity (PD) (Devictor et al. 2010). If communities exhibiting varying levels of these diversity components are situated in distinct locations, regions with high TD might not support elevated FD levels or a wide range of PD (Faith 1992, Mason et al. 2005, Monnet et al. 2014). This gap in understanding hinders effective policy responses aimed at addressing the underlying causes of biodiversity declines (Larsen et al. 2024).

Protected areas (PAs) play a crucial role in global biodiversity conservation and are central to international strategies aimed at mitigating species extinction rates (Geldmann et al. 2013, Barnes et al. 2016). Over the past decade, numerous studies have examined the taxonomic dynamics of bird communities in protected areas (Baselga et al. 2015, Low et al. 2024). However, research focusing on the taxonomic, functional and phylogenetic diversity of species remains limited. For example, a shift in bird community dynamics over 35 years in an undisturbed Amazonian rainforest showed a decrease in insectivorous species and an increase in frugivorous species (Stouffer et al. 2020). Such biodiversity losses in undisturbed forests indicate hidden declines, which may be widespread across conserved tropical forests and other intact ecosystems (Stouffer et al. 2020). Furthermore, Oliveira and dos Anjos (2022) proved that temporal beta diversity in birds is dominated by taxonomic turnover. The absence of significant changes in functional diversity implies functional redundancy amongst the changed species (Oliveira and dos Anjos 2022). While it is possible to assess the impacts of bird losses at species level, the overall impact of species replacements on ecosystem functionality remains unknown. Key questions include the extent of this phenomenon in other protected areas and whether the colonising species are effectively taking over the ecological roles of those that have been lost (Oliveira and dos Anjos 2022). If replacement species share similar functional characteristics, the ecological role in a community can be maintained even if they are lost or replaced (Oliveira and dos Anjos 2022). However, it remains uncertain whether the changes in the richness of functionally correlated species affect phylogenetic diversity (Webb 2000, Mason et al. 2005, Miller et al. 2018).

China is recognised as a mega-diverse country (Li et al. 2016) and the conservation of flora and fauna is critically reliant on China's network of PAs. Currently, China has 2,729 PAs that serve as the cornerstone of the country's conservation framework (Li et al. 2016). Birds are an important part of the global ecosystem. Bird diversity not only reflects the health of ecosystems, but is also a key indicator for evaluating the effectiveness of protected areas (Luck et al. 2012). Numerous studies have mainly focused on cataloguing the biodiversity of PAs in China and these data provide valuable information for PAs (Li et al. 2016, Wang et al. 2022, Wu et al. 2023). However, previous studies on bird diversity in protected areas have mainly focused on taxonomic diversity, relying on short-term monitoring (He et al. 2018, Wang et al. 2022). Collecting biodiversity time-series data is costly in terms of time, money and manpower (Balmford et al. 2009). Additionally, management agencies frequently lack the capacity or willingness to undertake such extensive monitoring due to budgetary limitations (Geldmann et al. 2013). From the perspective of species conservation, long-term ecological studies are essential for identifying changes in various aspects of biodiversity and evaluating their impact on the ecological functions provided by communities (Oliveira and dos Anjos 2022).

In this study, we monitored bird communities for nine years (2014-2022) from the Qiyunshan National Nature Reserve, China, to examine interannual changes in taxonomic diversity, functional diversity and phylogenetic diversity. We address the following questions: (i) Is there an increasing or decreasing trend in bird diversity in PAs over a short period and are these trends consistent across the three diversity metrics, i.e. the asynchrony of taxonomic, functional and phylogenetic diversity, in birds (Monnet et al. 2014, Miller et al. 2018)? (ii) are the ecological roles of bird communities being maintained in a protected area where temporal taxonomic changes are occurring?

## Methods and materials

### Study area

**Figure 1.**
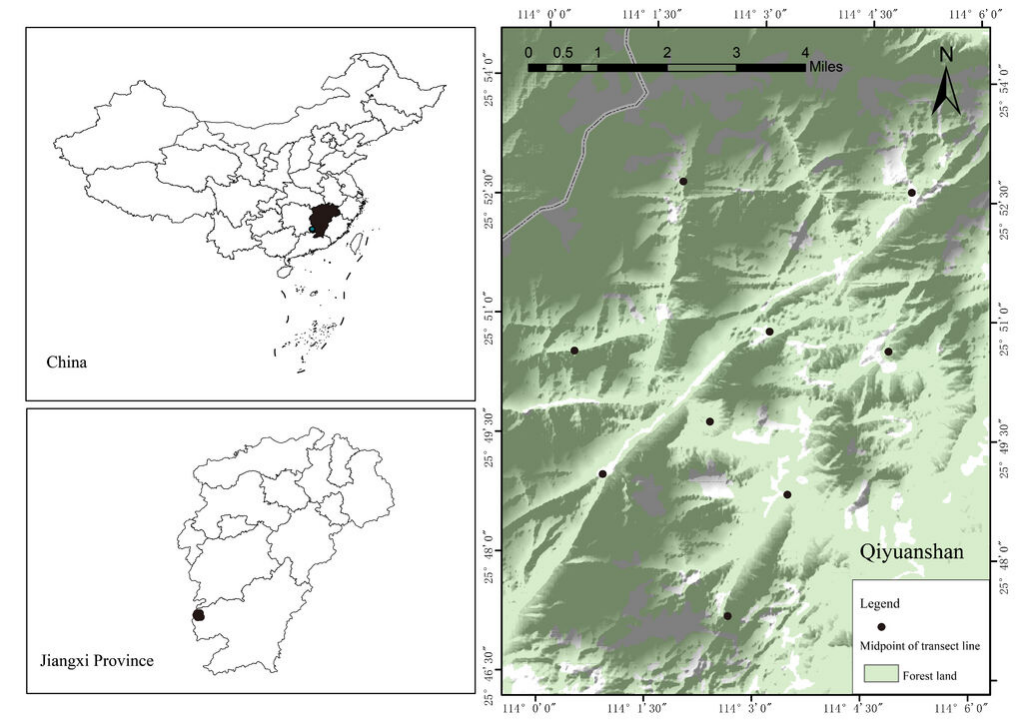
Location of the study area.

The study was carried out in the Qiyunshan National Nature Reserve (Qiyunshan) located in Jiangxi Province, China (113°54'-114°07' E, 25°41'-25°54' N) (Fig. 1). Officially established as a protected area in 1997, cover a total area of 17,105 ha with a remarkable forest coverage rate of 97.6% (Liu et al. 2010). The Reserve features a humid subtropical monsoon, characterised by a warm climate and abundant rainfall. The average annual precipitation is 1,750 mm and the average temperature is 17℃ (Liu et al. 2010, Qian et al. 2022). The minimum altitude of the Reserve is 300 m and the maximum is 2,061.3 m. Vegetation types include evergreen broadleaved forest, mixed coniferous broadleaved forest and meadow (Huang et al. 2020, Qian et al. 2022). Over the past decade, there have been no notable changes in land use within Qiyunshan, due to the stringent protection measures implemented by the Chinese government. Despite its ecological importance, biodiversity studies on Qiyunshan remain limited. Most of the studies have focused on migratory birds (Huang et al. 2008, Liu et al. 2010), with relatively few studies focusing on mammals (Qian et al. 2022) and butterfly diversity (Huang et al. 2020).

### Bird surveys

Bird monitoring in Qiyunshan was conducted annually from 2014 to 2022 using the line transect method (Bibby et al. 1992). Ten transects, each measuring 2-4 km in length, were established, spaced 200-500 m apart from each other. The number of transects, along with their starting and ending points, remained constant for all survey years (Fig. 1). The bird observations were carried out by trained ornithologists, with two survey periods annually, the first from 1 April to 1 May and the second observation period from 1 June to 30 June. The bird surveys were conducted in the morning from 07:00 h to 10:00 h and from 17:00 h to 19:00 h, aligning with bird activities using binoculars. Bird species and the number of individuals were recorded within 50 m on both sides of transects. Surveys were not conducted during unfavourable weather conditions, such as rain or heavy fog. The identification of bird species was based on Zheng (2023). We referred to the List of State Key Protected Wild Animals in China (http://www.forestry.gov.cn) and the International Union for Conservation of Nature (IUCN) Red List of Threatened Species (http://www.iucnredlist.org) to identify the protection levels and threat levels of birds. Only three observers conducted all sampling events to ensure standardisation of accuracy in species detection.

### Alpha diversity

**Figure 2.**
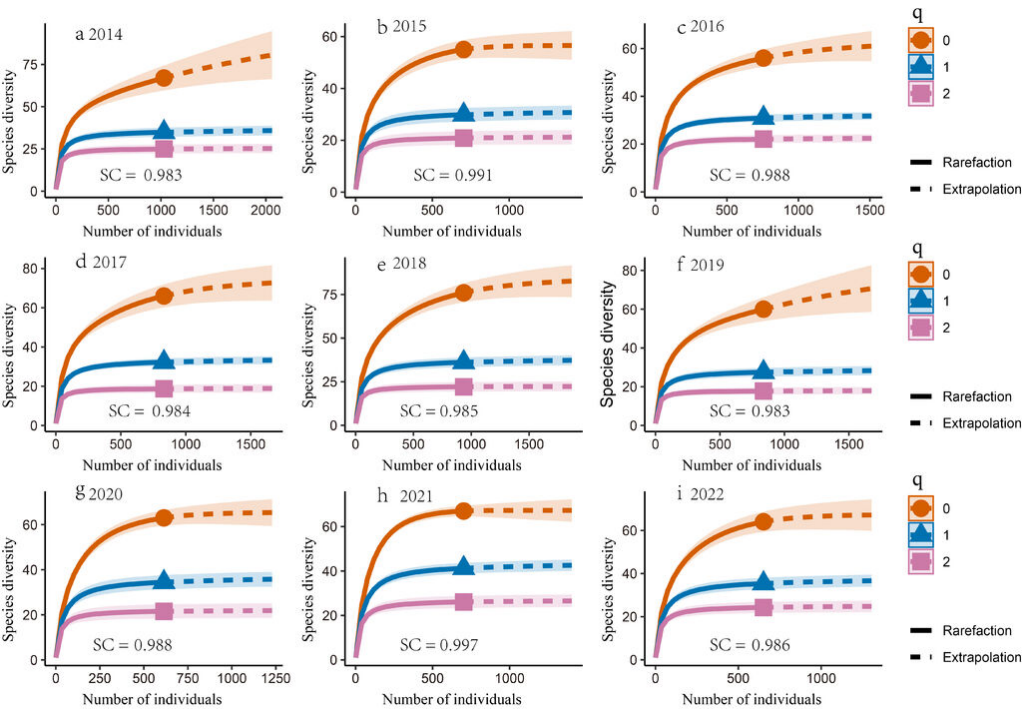
The rarefaction and extrapolation of bird species diversity in Qiyunshan were measured using the Hill number in various years (2014-2022). The solid lines represent rarefaction, while the dashed lines represent extrapolation; shaded regions, 95% confidence intervals; " q = 0" represents species richness; "q = 1" represents the Shannon-Wiener Index reflecting common species; "q = 2" represents the Simpson Index indicating dominant species; SC represents sample coverage. **a** 2014; **b** 2015; **c** 2016; **d** 2017; **e** 2018; **f** 2019; **g** 2020; **h** 2021; **i** 2022.

To evaluate the trend of taxonomic diversity, we assessed the adequacy of bird sampling using the 'iNEXT' package in R. This allowed us to generate rarefaction and extrapolation curves, based on sample size and coverage (Chao et al. 2014, Li et al. 2024). A sample coverage exceeding 0.90 indicates sufficient sampling (Li et al. 2024) (Fig. 2). We assessed bird diversity through species richness, Shannon-Wiener Diversity Index and Simpson Index following Hill numbers (Li et al. 2024), where "q = 0" represents species richness, "q = 1" represents the Shannon-Wiener Index reflecting common species and "q = 2" represents the Simpson Index indicating dominant species (Chao et al. 2014, Li et al. 2024).

To quantify functional diversity, we obtained data on six functional traits from Wang et al. (2021) that characterise the niche of the species, offering insights into how species utilise and compete for resources within their habitats (Luck et al. 2012, Li et al. 2024). We used the following traits: body mass, beak length, body length, wing length, feeding guild (insectivores, carnivores, nectarivorous, granivores and omnivores) and nest site (ground, water, scrub, canopy, rock wall). Amongst these traits, body mass, beak length, body length and wing length were continuous variables (Li et al. 2024). The feeding guild and the nest site were categorical variables. Due to computational limitations, we converted categorical variables into ordinal variables. Four functional diversity metrics were calculated: functional richness (FRic), evenness (FEve), divergence (Fdiv) and dispersion (FDis) (Oliveira and dos Anjos 2022). Functional diversity is defined as follows (Mason et al. 2005, Villéger et al. 2008, Mouchet et al. 2010, Laliberté and Legendre 2010, Li et al. 2024): functional richness measures the extent of functional space utilised by a group of species. Functional evenness indicates the uniformity of species abundances across functional space (Li et al. 2024). Functional divergence quantifies the distance between high species abundances and the centre of functional space (Li et al. 2024). The three indices complement each other. Additionally, functional divergence, functional evenness and functional dispersion are not influenced by species richness, making it possible to compare communities with varying species richness without bias (Villéger et al. 2008, Li et al. 2024). Functional dispersion measures the average distance of each species to the centre of all species, taking into account their respective weights (Li et al. 2024). The analysis of functional diversity was conducted in the R "fundiversity" package (R Core Team 2024).

To perform phylogenetic analyses, we used a vector of scientific species names to generate a phylogeny from megatrees (see details in Li (2023) and Li et al. (2024)). Based on the bird species checklist from our survey, we constructed a phylogenetic tree by classifying species into orders, families and genera using supertrees (Li et al. 2024) (Suppl. material 1). We constructed a phylogenetic tree using the BirdTree database (http://birdtree.org) (Jetz et al. 2012, Li 2023, Li et al. 2024). This phylogenetic tree enables the calculation of Faith’s index of phylogenetic diversity (Faith 1992), mean pairwise distance (MPD) and mean nearest taxon distance (MNTD) for analysis of phylogenetic diversity (Webb 2000). To quantify phylogenetic patterns in community structure, we used a null model with an independent swapping algorithm to stochastically generate species richness and occurrence frequencies (Li et al. 2024). Subsequently, mean FD, MPD and MNTD values for the null model are calculated and compared with the observed values (Li et al. 2024). The standard effect size (SES) is calculated using the following formula:

SES=（*M_obs_* - *M_null_*）/ *SD_null_*

where *M_obs_* is the observed value of FD/MPD/MNTD, *M_null_* is the average of the 999 null model FD/MPD/MNTD values generated randomly and *SD_null_* is the standard deviation of the 999 random values (Li et al. 2024). A negative SES.MPD/MNTD indicates a clustered community phylogenetic structure, whereas a positive SES suggests an overdispersion structure (Webb 2000, Li et al. 2024). SES.MPD or MNTD greater than 1.96 (*p* < 0.05) signifies a significant overdispersion community structure, potentially attributed to competitive exclusion (Li et al. 2024). Conversely, an SES less than -1.96 (*p* < 0.05) indicates a significantly clustered community structure, likely influenced by environmental filtering. An SES falling within the range of -1.96 to 1.96 (*p* > 0.05) implies that the community assembly follows a random process (Webb 2000, Li et al. 2024). The analysis was conducted using the R package "picante" (R Core Team 2024).

### Beta diversity

Beta diversity reflects two distinct ecological processes, spatial species turnover and nestedness of assemblages (Baselga 2010), which result from two antithetic processes, namely species replacement and species loss, respectively (Baselga 2010). This method also allows for the computation of dissimilarity between locations over time 1 and time 2 (Baselga et al. 2015). We computed the dissimilarity of bird composition for Qiyunshan between 2014 and 2022, considering the turnover and nestedness components of temporal change and the sum of both values (overall change) (Baselga 2010). Following this method, we utilised the Sørensen pairwise dissimilarity index for evaluating the overall beta diversity (*β_sor_*) (Zhao et al. 2021). Then, we employed the Simpson dissimilarity index to quantify the turnover (*β_sim_*) and nestedness (*β_sne_*) components (Baselga 2010, Baselga 2012). Pairwise and multiple-site dissimilarity partitioning frameworks were performed in R using the "betapart" package (Baselga and Orme 2012). To strengthen our results more robustly, we used temporal beta diversity (TBI) to measure species composition differentiation through time (Legendre 2019). The temporal beta diversity was divided into losses and gains of species independently for each site to ascertain the direction of change (Legendre 2019, Jiménez et al. 2024). TBI analysis is especially interesting in species-rich communities where we cannot examine the changes in every species individually (Legendre and Condit 2019). We used the "%difference" method to compute the percentage difference index, this index also being known as the Bray-Curtis dissimilarity (Legendre 2019). Temporal beta diversity was carried out in the "adespatial" package in R (R Core Team 2024).

### Data analysis

The non-parametric Mann-Kendall test was used to assess the monotonic trend in the time series of bird diversity for alpha diversity metrics. The non-parametric Mann-Kendall test is commonly employed to detect monotonic trends in a series of environmental data. To explore the potential non-linear trends in bird diversity over the past nine years in Qiyunshan, Local Polynomial Regression was fitted to the alpha diversity metrics. The Mann-Kendall test and Local Polynomial Regression were performed in R using the "trend" package (R Core Team 2024). Generalised Dissimilarity Modelling (GDM) was used to estimate beta diversity across multiple sites (Ferrier et al. 2007). GDM is a powerful and unique method for characterising and predicting beta diversity (Mokany et al. 2022), the change in biodiversity over space, time and environmental gradients (Mokany et al. 2022). GDM uses I-spline basis functions to transform each of the predictor variables (Mokany et al. 2022), so that summed absolute difference in the transformed predictor values (Ferrier et al. 2007, Mokany et al. 2022), combined with the model intercept, provide a predicted ecological distance. The negative exponential link function ensures predicted dissimilarities increase and saturate with increases in the predicted ecological distance (Ferrier et al. 2007, Mokany et al. 2022). Since nestedness is not a central issue in our study, we considered the overall beta diversity, turnover components and temporal beta diversity. GDM was carried out in the "gdm" package in R (R Core Team 2024).

## Results

**Figure 3.**
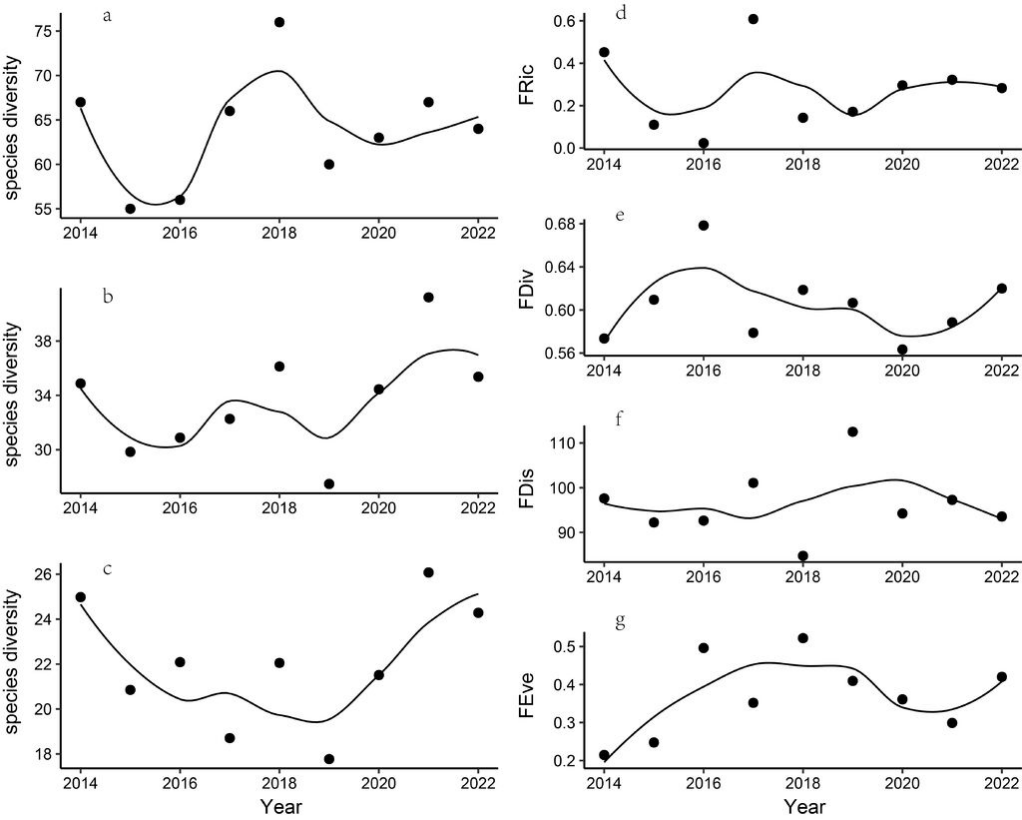
Annual trends in taxonomic diversity and functional diversity of birds. **a** "q = 0" represents species richness; **b** "q = 1" represents the Shannon-Wiener Index reflecting common species; **c** "q = 2" represents the Simpson Index indicating dominant species; **d** Functional richness (FRic); **e** Functional divergence (FDiv); **f** Functional dispersion (FDis); **g** Functional evenness (FEve).

**Figure 4.**
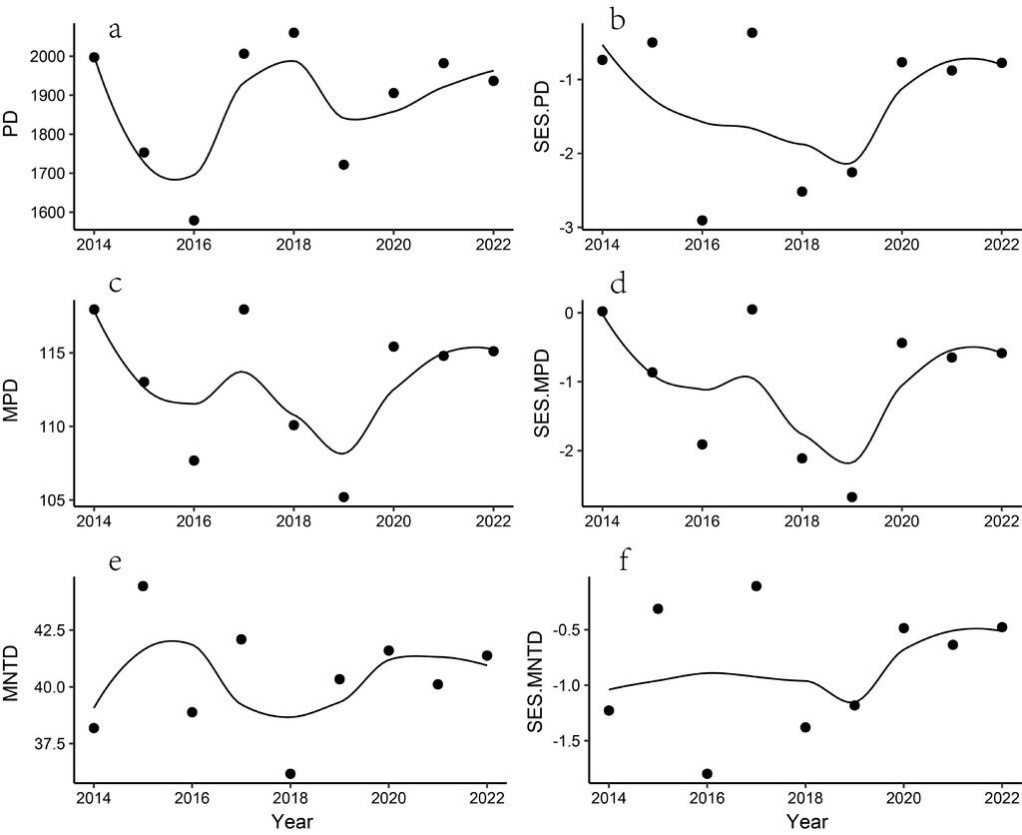
Annual trends in phylogenetic diversity of birds. **a** phylogenetic diversity (PD); **b** standard effect size of PD (SES.PD); **c** mean pairwise distance (MPD); **d** standard effect size of MPD (SES.MPD); **e** mean nearest taxon distance (MNTD); **f** standard effect size of MNTD (SES.MNTD).

We recorded a total of 118 bird species from 2014 to 2022, with the dominant species including Chestnut Bulbul (*Hemixoscastanonotus*), Grey-cheeked Fulvetta (*Alcippemorrisonia*), Great Tit (*Parusmajor*), Mountain Bulbul (*Ixosmcclellandii*) and Chinese Barbet (*Psilopogonfaber*) (Suppl. material 1). Additionally, we recorded two species listed as national first-class protected species and fourteen species listed as national second-class protected species in China. Yellow-breasted Bunting (*Emberizaaureola*) is listed as Critically Endangered (CR) and White-eared Night Heron (*Gorsachiusmagnificus*) is listed as Endangered (EN) in the IUCN Red List of Threatened Species. Our analysis of various diversity metrics revealed that bird species richness (*z* = 0.628, *p* = 0.529), the Shannon-Wiener Index (*z* = 1.147, *p* = 0.529), the Simpson Index (z = 0.104, *p* = 0.917), functional richness (*z* = 0.521, *p* = 0.602), phylogenetic diversity (*z* = 0.104, *p* = 0.917), functional dispersion (*z* = 0.104, *p* = 0.917), functional evenness (*z* = 0.938, *p* = 0.348), functional divergence (*z* = 0.104, *p* = 0.917), mean pairwise distance (MPD) (*z* = 0.104, *p* = 0.917), mean nearest taxon distance (MNTD) (*z* = 0.104, *p* = 0.917), SES.PD (*z* = 0.104, *p* = 0.917), SES.MPD (*z* = -0.313, *p* = 0.755) and SES.MNTD (*z* = 0.729, *p* = 0.466) revealed no significant increases or decreases. All diversity metrics showed non-linear trends (Figs 3, 4). Our findings indicate that the composition of bird communities is shaped by environmental filtering and neutral processes (Fig. 4).

**Figure 5.**
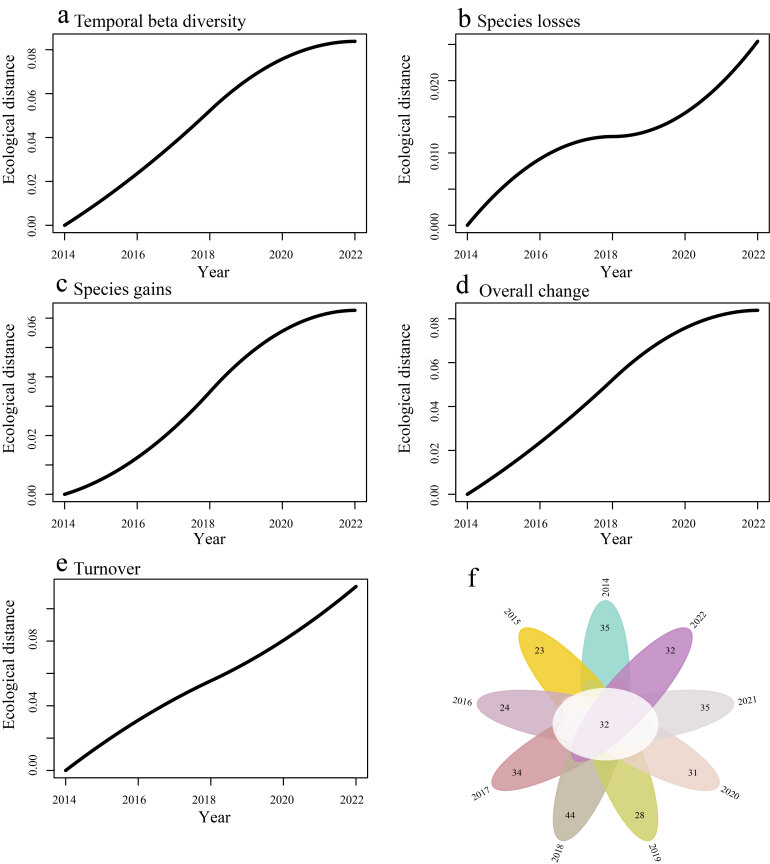
Temporal beta diversity of birds. **a** temporal beta diversity; **b** species losses; **c** species gains; **d** overall change; **e** turnover; **f** number of shared species in different years.

Our findings indicate that temporal beta diversity, species gains and overall beta diversity all gradually increased and then stabilised during the study period (Fig. 5). In contrast, the loss and turnover of species showed a sharp upward trend. Furthermore, for overall beta diversity (*β_sor_* = 0.569), we found that the turnover component (*β_sim_* = 0.507) is greater than the nestedness component (*β_sne_* = 0.06). This indicates that changes in overall beta diversity were primarily driven by the turnover component. In addition, the number of shared species varies greatly in different years (Fig. 5).

## Discussion

### Changes in taxonomic, functional and phylogenetic diversity

Consistent with studies in the tropical forest nature reserve (Stouffer et al. 2020, Oliveira and dos Anjos 2022), our study showed taxonomic changes in bird communities in Qiyunshan over time. We identified taxonomic turnover as the primary mechanism driving annual variations in species composition. However, despite variations in species richness and composition, both FD and PD remained stable throughout the study period. This non-linear pattern highlights the dynamic nature of bird communities, where temporal shifts in species co-existence, resulting in fluctuations in species richness and composition, even in undisturbed nature reserves (Oliveira and dos Anjos 2022). Our findings suggest that protected areas like Qiyunshan have the potential to attract novel bird species that share similar functional and genetic relationships with existing communities. This dynamic contributes to functional redundancy amongst species, ensuring that FD and PD remain largely unaffected despite taxonomic changes (Villéger et al. 2008, Mouchet et al. 2010, Oliveira and dos Anjos 2022). For instance, we documented Asian Barred Owlet (*Glaucidiumcuculoides*) in 2014, 2018 and 2019 and we recorded Collared Owlet (*Glaucidiumbrodiei*) from 2018 to 2022. Although these two carnivorous birds were recorded in different years, their similar functional traits and phylogenetic relationship prevented any niche gap. Furthermore, no significant trends were observed in bird taxonomic composition (Suppl. material 2), indicating that the overall taxonomic structure remained stable.

Furthermore, we observed no distinct boundary between the forested areas within and outside Qiyunshan. This lack of separation, combined with birds' strong dispersal ability enables them to effectively colonise and move across reserves, facilitating their widespread distribution (Tanentzap and Lloyd 2017). Surprisingly, we observed that the species loss showed a sharp upward trend. This phenomenon may be attributed to spillover effects, which amplify the conservation benefits by facilitating species dispersion beyond the boundaries of the protected area. For example, the spillover effects of fenced reserves in New Zealand, where avian species breed within the reserve and disperse into surrounding areas demonstrate this process (Tanentzap and Lloyd 2017). Since we do not have data on birds around Qiyunshan, our conclusion should be interpreted cautiously. Policy-makers and conservation authorities should consider these limitations when applying our findings to broader conservation strategies.

Our findings indicate that the patterns observed in all measures of biodiversity are not always consistent. Numerous studies have demonstrated the asynchrony of TD, FD and PD in birds (Villéger et al. 2008, Devictor et al. 2010, Morelli et al. 2017). For example, breeding birds in France from 1989 to 2012 showed noticeable variability both within and amongst the temporal trends of each aspect of bird diversity, revealing a notable increase in TD and PD (Monnet et al. 2014), while FD showed no discernible pattern (Monnet et al. 2014). Similarly, in China, water-bird species richness and SES.MNTD declined from the 1950s to the 2010s without significant trends (Che et al. 2021), whereas SES.MPD and FD experienced a significant decline during the same period (Che et al. 2021). Previous studies have shown that PD is a better measure of biodiversity than taxonomic counts (Miller et al. 2018). PD is particularly valuable when many traits exhibit a phylogenetic signal, indicating that PD can assess the functional trait space of a community (Srivastava et al. 2012). However, the effectiveness of PD relies on the robustness of phylogenetic signals to both traits and interactions (Srivastava et al. 2012). If future studies can integrate these intricacies, there is potential for conceptual unification of evolutionary biology and ecosystem ecology through exploring the relationships between PD and ecosystem function (Srivastava et al. 2012).

### Community assembly

Our findings indicate that the composition of bird communities is shaped by both environmental filtering and neutral processes. For community assembly rules, phylogenetic clustering may result from the local extinction of species that are phylogenetically dissimilar to those present or the colonisation of species that are phylogenetically and functionally similar to the residents (Li et al. 2015). However, according to the neutral theory of biodiversity, ecological communities are dynamic and open systems where species co-occurrence is unstructured (Chave 2004, Hubbell 2011, Oliveira and dos Anjos 2022). Communities are randomly structured and independent with an assembly process driven by neutral dispersal, local stochastic extinction and ecological drift, rather than species-specific characteristics (Hubbell 2011, Oliveira and dos Anjos 2022).

Previous studies suggest that PD and MNTD are effective metrics for detecting habitat filtering, while MPD is more suited to identifying competitive exclusion (Miller et al. 2016). For community assembly, our findings are consistent with previous studies in the Ailao Mountains in southwest China (He et al. 2018), Nyungwe National Park in southwest Rwanda (Rurangwa et al. 2021) and Mt. Kenya of Kenya (Gao et al. 2024). Environmental filtering suggests that only species with similar traits can survive and reproduce in a given abiotic environment (Miller et al. 2016). Thus, if these traits are evolutionarily conserved, habitat selection will lead local communities that are more phylogenetically similar than expected by chance, based on phylogenetic clustering (Miller et al. 2016). However, long-term patterns of plant phylogenetic diversity have indicated a decrease in phylogenetic clustering as succession progresses, with colonisation playing a primary role in driving this transition (Li et al. 2015). Early colonists were closely related and functionally similar to resident species, whereas later colonists showed decreasing similarity to the existing species (Li et al. 2015). In this study, the phylogenetic clustering pattern may be influenced by the resource availability of Qiyunshan. The lack of clear patterns of the phylogenetic assemblage structures suggests complex interactions between ecological and evolutionary processes (Gao et al. 2024).

### Temporal beta diversity

Understanding the temporal dynamics of biodiversity is essential for developing effective environmental management policies in PAs. Our findings indicate a significant increase in temporal beta diversity, with the turnover component being larger than the nestedness component, suggesting potential changes in bird community composition in PAs. Our findings are consistent with previous studies in southwest France (Baselga et al. 2015) and the Atlantic Forest protected area in southern Brazil (Oliveira and dos Anjos 2022). Generally, temporal changes in species composition may result from deterministic processes driven by environmental changes, random processes of colonisation and local extinction (Baselga et al. 2015). No significant land-use changes were observed in Qiyunshan during the study period. Even in anthropogenic landscapes, prior studies have demonstrated that alterations in land cover within agricultural environments have had minimal effects on the temporal beta diversity of bird assemblages (Baselga et al. 2015). More broadly, previous studies suggest that stochastic processes may play a role in explaining temporal changes in biodiversity (Oliveira and dos Anjos 2022) and that bird species appeared and disappeared from specific localities in a random-like way (Baselga et al. 2015). In other words, this phenomenon likely reflects random variation driven by the observed random losses and gains of species (Legendre 2019, Legendre and Condit 2019).

## Conclusions

In conclusion, the species composition of the studied bird community varied significantly over time. Our findings highlight that protected areas can maintain functional and phylogenetic structures despite species turnover, suggesting functional redundancy amongst changing species. In China, nature reserves typically prioritise stabilising and increasing species diversity as a key conservation goal. We suggest that functional and phylogenetic diversity be considered essential indicators alongside species richness when evaluating conservation outcomes in nature reserves. This approach provides a more comprehensive reflection of ecological community complexity and provides information for more effective conservation strategies. However, the persistent increase in species loss over time raises concerns about its potential impact on the future functional stability of the system. Future research should investigate species dynamics inside and outside protected areas as well as their responses to environmental changes and human activities.

## Supplementary Material

A81809B9-BE1B-5F1D-AA49-E1993BC8D2C7 10.3897/BDJ.13.e145093.suppl1 Supplementary material 1 List of species Data type Table Brief description List of bird species in Qiyunshan National Nature Reserve. File: oo_1200918.xls https://binary.pensoft.net/file/1200918 Binqiang Li

6E1F4C13-5402-5F11-9A29-89B3ED0A667B 10.3897/BDJ.13.e145093.suppl2 Supplementary material 2 Taxonomic unit (orders and families) Data type Table Brief description Taxonomic unit (orders and families) changes of birds in Qiyunshan National Nature Reserve. File: oo_1268134.xlsx https://binary.pensoft.net/file/1268134 Binqiang Li
